# Performance of a new hand-held device for exhaled nitric oxide measurement in adults and children

**DOI:** 10.1186/1465-9921-7-67

**Published:** 2006-04-20

**Authors:** K Alving, C Janson, L Nordvall

**Affiliations:** 1 Department of Physiology and Pharmacology, Karolinska Institutet, SE-17177 Stockholm, Sweden; 2 Department of Medical Sciences, Uppsala University, Uppsala, Sweden; 3 Department of Women's and Children's Health, Uppsala University, Uppsala, Sweden

## Abstract

**Background:**

Exhaled nitric oxide (NO) measurement has been shown to be a valuable tool in the management of patients with asthma. Up to now, most measurements have been done with stationary, chemiluminescence-based NO analysers, which are not suitable for the primary health care setting. A hand-held NO analyser which simplifies the measurement would be of value both in specialized and primary health care. In this study, the performance of a new electrochemical hand-held device for exhaled NO measurements (NIOX MINO) was compared with a standard stationary chemiluminescence unit (NIOX).

**Methods:**

A total of 71 subjects (6–60 years; 36 males), both healthy controls and atopic patients with and without asthma were included. The mean of three approved exhalations (50 ml/s) in each device, and the first approved measurement in the hand-held device, were compared with regard to NO readings (Bland-Altman plots), measurement feasibility (success rate with 6 attempts) and repeatability (intrasubject SD).

**Results:**

Success rate was high (≥ 84%) in both devices for both adults and children. The subjects represented a FE_NO _range of 8–147 parts per billion (ppb). When comparing the mean of three measurements (n = 61), the median of the intrasubject difference in exhaled NO for the two devices was -1.2 ppb; thus generally the hand-held device gave slightly higher readings. The Bland-Altman plot shows that the 95% limits of agreement were -9.8 and 8.0 ppb. The intrasubject median difference between the NIOX and the first approved measurement in the NIOX MINO was -2.0 ppb, and limits of agreement were -13.2 and 10.2 ppb. The median repeatability for NIOX and NIOX MINO were 1.1 and 1.2 ppb, respectively.

**Conclusion:**

The hand-held device (NIOX MINO) and the stationary system (NIOX) are in clinically acceptable agreement both when the mean of three measurements and the first approved measurement (NIOX MINO) is used. The hand-held device shows good repeatability, and it can be used successfully on adults and most children. The new hand-held device will enable the introduction of exhaled NO measurements into the primary health care.

## Background

Since the original reports of the presence of nitric oxide (NO) in exhaled breath of mammals including humans [1], and of the increased levels in subjects with asthma [2], there has been a rapidly increasing interest in the measurement of exhaled NO. The concentration of NO in exhaled breath relates to the degree of eosinophilic inflammation in the airways [3-5], and NO measurement has been shown to be a valuable tool both to diagnose [6,7] and to monitor the therapy of patients with asthma [8,9].

Up to now, a single type of NO analyser has been used for nearly all measurement of exhaled NO: the chemiluminescence NO analyser [10]. These instruments are based on a technology developed in the 1970's [11] and were originally used for environmental and atmospheric analyses. The chemiluminescence-based NO analysers are fast-responding, highly sensitive (detection limit 1 parts per billion (ppb) or lower) and specific for NO gas. However, they are also rather bulky and expensive, and they need to be calibrated on site, drawbacks that have been limiting factors for the introduction of exhaled NO measurements in routine clinical work. An alternative would be to use electrochemical sensors, but they have not been sensitive enough for analysis of NO in the low ppb range. Recently, however, a new electrochemical sensor has been developed, based on the amperometric technique (the production of a current when a potential is applied between two electrodes), which is suitable for NO analysis in exhaled breath [12]. This sensor has been incorporated into a hand-held measuring device that complies with international guidelines for exhaled NO measurements [13].

In this study, the new hand-held device was compared to a chemiluminescence-based stationary device for exhaled NO measurements that has previously proven to provide repeatable results [14]. Both these instruments are now cleared for clinical use in Europe and the stationary device has also been cleared by the US Food and Drug Administration [15]. The two devices were compared with regard to NO readings, measurement repeatability and feasibility, in a sample of 71 children and adults with and without asthma.

## Methods

### Subjects

Subjects were consecutively recruited at two separate clinics (adult and pediatric) at Uppsala University Hospital. A total of 75 subjects were invited; 34 adults (6 males, 38 ± 11 years; mean ± SD) and 41 children (30 males, 12 ± 3 years). The total age range was 6–60 years. Twentyone subjects were non-atopic healthy controls, 52 subjects were atopic and 39 subjects had a diagnosis of asthma. None of the subjects had used any of the NO instruments over the preceding 6 months and were thus considered unexperienced with NO measurements. Subjects with a FE_NO _(fraction of expired NO) value of < 8 ppb were excluded (tested with the chemiluminescence-based instrument). At the time of the study, the detection limit for the hand-held device was considered to be 8 ppb. This has later been corrected by the manufacturer (see Table 1). The study was approved by the regional ethics committee and all subjects gave written informed consent.

**Table 1:** Device comparison. Some characteristics that are not identical in the two devices are given. Most other characteristics are similar, for example NO scrubbing of inhaled air and the exhalation flow control.

	NIOX	NIOX MINO
Measurement range (ppb)	1.5–200	5–300
Accuracy	< 50 ppb: ± 2.5 ppb ≥ 50 ppb: ± 5%	< 50 ppb: ± 5 ppb ≥ 50 ppb: ± 10%
Visual feedback	Computer screen	Display via mirror
Calibration on site	Yes	No
Dimensions (cm)	50 × 30 × 40	24 × 13 × 10
Weight (kg)	40	0.8

### Experimental protocol

**Figure 1:**
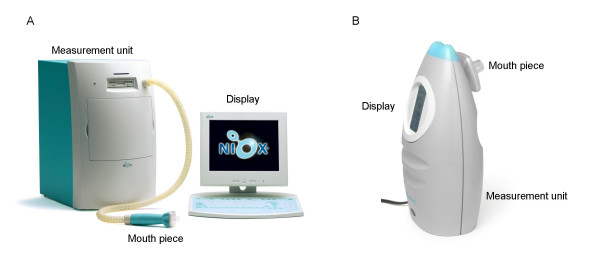
**Appearance of devices**. Illustrations of (A) the NIOX and (B) the NIOX MINO. Note that the relative size is not proportional (see Table 1 for device dimensions).

Under guidance of clinical personnel, all subjects inhaled NO-free air (built-in NO scrubbers) to close to total lung capacity and exhaled during 10 s at a flow rate of 50 ml/s to provide three approved FE_NO _measurements in each of the two devices (NIOX^® ^Nitric Oxide Monitoring System (NIOX) and NIOX MINO^® ^Airway Inflammation Monitor (NIOX MINO); Aerocrine AB, Solna, Sweden). Oral pressure was measured and the subjects were instructed to keep the pressure between 12–18 cmH_2_O in both devices with the help of visual feedback (provided via a mirror in NIOX MINO). Exhalation flow rate was kept at 50 ± 5 ml/s with calibrated dynamic flow resistors in both devices. In the NIOX, the mean NO concentration over the last 3 s of exhalation is calculated and the NO plateau is evaluated by linear regression, whereas in the NIOX MINO, the last 3-s portion of exhaled air is led into the measurement chamber containing the sensor. Analysis takes 100 s before a result representing the NO concentration in this mixed gas portion is presented. Sampling technique in both devices complies with current international guidelines [13]. The instruments are further described in Fig 1 and Table 1.

Measurements were performed in randomized device order (at most 6 attempts per device). The mean of three measurements in each device, or the first approved measurement in the NIOX MINO were used for agreement studies. After these measurements, subjects also attempted one valid FE_NO _measurement (at most 3 attempts) in the hand-held device in a simulated home-use environment where each subject performed the FE_NO _measurement without the assistance of clinical personnel.

### Statistics

Data are presented as arithmetic mean ± standard deviation (SD), or median [interquartile range] when appropriate. For comparison between devices, intraclass correlation coefficients (ICCs) were calculated and presented as reliability coefficients, and Bland-Altman plots were constructed. Repeatability was calculated from intrasubject SD. Student's paired t-test was used to compare the mean number of attempts in the two devices. The success rate was calculated as the proportion of subjects succeeding in obtaining three valid FE_NO _measurements out of a maximum of six attempts in each device, or one successful measurement out of a maximum of three attempts using the NIOX MINO in the simulated home use. Differences in success rate was evaluated by Fisher's exact test.

## Results

### Success rate for approved measurements

Four subjects out of 75 had FE_NO _measurements < 8 ppb and were excluded from the study. They were all younger children (age ≤ 13 years). Of all subjects (n = 71) who made an attempt to use the NIOX or the NIOX MINO under guidance of clinical personnel, only a few failed to obtain three approved FE_NO _measurements out of a maximum of 6 attempts (Table 2). These were primarily younger children (age ≤ 13 years) who failed when attempting to use the NIOX MINO (6 out of 7). The study subjects were similarly successful in a simulated home-use environment using the NIOX MINO where subjects were to obtain one approved measurement out of three attempts without guidance. There was no significant difference in success rate between the NIOX and the NIOX MINO, or between the clinical setting and the simulated home use, except for children being slightly less successful than adults when attempting to use the NIOX MINO (p < 0.05, Fisher's exact test).

**Table 2:** Success rate. Fraction of patients (%) that were able to achieve three accepted measurements out of at most 6 attempts under guidance (clinical setting), or one accepted measurement out of at most 3 attempts without guidance (simulated home use).

Mode	Clinical setting		Home use
Instrument	NIOX	NIOX MINO	NIOX MINO

All subjects (n = 71)	94	92	92
Children (n = 37)	95	84	84
Adults (n = 34)	94	100	100

In subjects that were successful in all three sets of measurements (n = 61), the mean number of attempts required to obtain three approved measurements was 3.8 ± 1.0 and 3.4 ± 0.8 for the NIOX and the NIOX MINO, respectively. The number of attempts was significantly lower for the NIOX MINO (p < 0.05; paired t-test). The mean number of attempts required by successful patients to obtain one approved measurement in the home-use environment was 1.1 ± 0.3.

Three adverse eventswere reported (mental stress, throat dryness, uncomfortable inhalation); they were all considered mild and were deemed unlikely to be caused directly by the study devices.

### Agreement between devices

**Figure 2:**
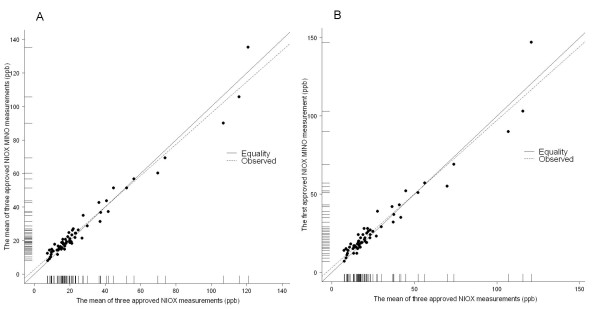
**Device agreement**. Scatter plots of the mean of three valid FE_NO _measurements using the NIOX vs (A) the mean of three valid FE_NO _measurements, or vs (B) the first valid FE_NO _measurement using the NIOX MINO (n = 61). Repeatability coefficients (ICCs) were (A) 0.97 and (B) 0.98, respectively (p < 0.001 for both).

**Figure 3:**
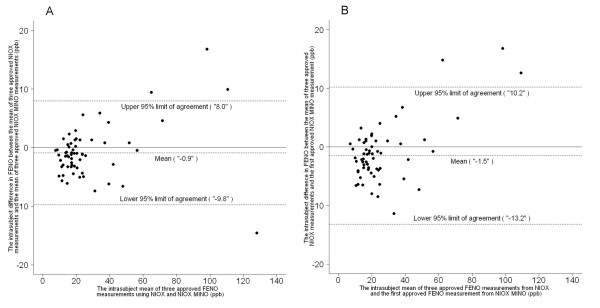
**Device agreement**. Bland-Altman plots of the mean of three valid FE_NO _measurements using the NIOX vs (A) the mean of three valid FE_NO _measurements, or vs (B) the first valid FE_NO _measurements using the NIOX MINO (n = 61).

The subjects represented a FE_NO _range of 8–147 ppb. The overall mean values for the NIOX and the NIOX MINO were 26.5 ± 24.2 ppb and 27.5 ± 23.2 ppb (n = 63 and 62, respectively). The reliability coefficient was high (r = 0.97) when comparing the individual mean values in the two devices (Fig 2A). The Bland-Altman plot shows agreement between the NIOX and the NIOX MINO when comparing the mean of three valid exhaled NO measurements (Fig 3A). The median of the intra-subject FE_NO _difference was -1.2 [-3.3, 0.8] ppb, suggesting that the NIOX MINO gave FE_NO _readings that were generally slightly higher than the FE_NO _measurements obtained using the NIOX. The 95% limits of agreement were -9.8 and 8.0 ppb, which indicates that for 95% of all subjects the difference between FE_NO _readings in NIOX and NIOX MINO is expected to lie in the interval [-9.8, 8.0] ppb. The Bland-Altman plot shows that the intrasubject FE_NO _difference increased with increasing FE_NO _level (Fig 3A).

In addition, we find the same degree of agreement between the NIOX and the NIOX MINO when comparing the mean of three approved exhaled NO measurements in the NIOX and the first approved measurement in the NIOX MINO in the clinical setting (Fig 2B, 3B). The median of the intra-subject FE_NO _difference was -2.0 [-4.0, 1.0) ppb, again suggesting that FE_NO _measurements with NIOX MINO were slightly higher than FE_NO _measurements using NIOX. The 95% limits of agreement were -13.2 and 10.2 ppb.

### Measurement repeatability

**Figure 4:**
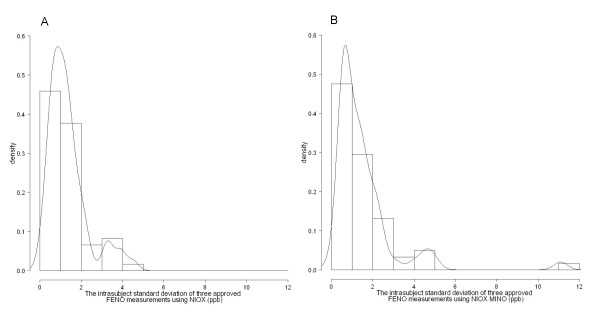
**Repeatability**. Histograms of the intra-subject SD of three valid FE_NO _measurements using (A) the NIOX (n = 63) and (B) the NIOX MINO (n = 62). The estimated distribution of SD is indicated with the line.

Repeatability was similar in the NIOX and the NIOX MINO. The 95^th ^percentile for the distribution of the repeatability (an estimate of the upper boundary of the repeatabilityfor 95% of all subjects) in the NIOX was 3.3 ppb compared to 4.6 ppb in the NIOX MINO. The median repeatability for NIOX and NIOX MINO was 1.1 [0.6, 1.6] and 1.2 [0.6, 2.0] ppb, respectively. The real and estimated distribution of intrasubject SDs are shown in Fig 4. One extreme observation concerning the repeatability in the NIOX MINO was noted (seen in Fig 4B). However, this observation was not treated as an outlier in the population.

## Discussion

Exhaled NO has been studied extensively over the past decade and reports of the clinical utility of this method in the management of patients with asthma are now appearing in the literature [8,9]. However, the introduction of the method into clinical routine has been restricted by the cost and complexity of existing NO analysers. In this study, the performance of a new hand-held device for exhaled NO measurements has been compared with that of a standard stationary unit.

When we compare the mean of three valid FE_NO _measurements using the established chemiluminescence-based NIOX and the NIOX MINO, which incorporates an electrochemical sensor, the results suggest clinically acceptable agreement between the two instruments. Measured FE_NO _levels obtained using the NIOX MINO were on average slightly higher than those obtained with the NIOX, and there was a tendency that the intrasubject FE_NO _difference increased with increasing FE_NO_. We believe that the difference between the two instruments is acceptable, considering the different measurement technologies and calibration procedures used in the two devices, and the results are in conformity with the declared accuracy for both the NIOX and the NIOX MINO. From a clinical point of view, accuracy will be more important in a FE_NO _range close to a cut-off between health and disease (20–35 ppb) than at higher FE_NO _levels. The NIOX MINO showed good agreement (within 95% limits of agreement) with the NIOX up to approximately 60 ppb, which indicates that the new hand-held device will be able to give clinical guidance with similar accuracy as the conventional chemiluminescence-based unit.

In general, the NIOX MINO and the NIOX had similar repeatability, except for one extreme observation with poor reproducibility in the NIOX MINO. However, this was seen in a subject with very high exhaled NO values (range 125–147 ppb in the NIOX MINO), and such variability at these high NO levels is of minor clinical importance. The repeatability agreed with the devices' technical specifications.

Success rates in achieving the required number of acceptable measurements were at least 84% for both devices and for both subject groups. Since all subjects were considered unexperienced with NO measurements, this indicates that both measurement techniques are generally well accepted by the patients. However, younger children failed slightly more frequently than adults when attempting to use the NIOX MINO. Interestingly, the number of attempts needed for successful subjects to achieve three acceptable measurements was significantly lower in the NIOX MINO compared to the NIOX. This could at least partly be explained by the fact that some measurements in the NIOX may be discarded after a linear regression analysis of the NO plateau has been performed, even though the number of regression failures was not recorded in the present study. The linearized plateau must not deviate more than 10% from the horizontal axis according to current guidelines [13]. In the NIOX MINO, the NO level in the last 3-s portion of mixed exhaled air is analyzed. Thus, the need for an analysis of the quality of a real-time NO plateau is avoided in the hand-held instrument.

Four subjects were excluded because of a low exhaled NO value (< 8 ppb). However, three of these subjects had a measurement above 5 ppb which is now the established lower detection limit of the NIOX MINO.

During the simulated home use, subjects were given the opportunity to use the NIOX MINO unassisted by study staff (children were assisted by their parents as they likely would be at home). This was performed after the clinical session, which would imitate what would normally happen, namely that the patient would receive training in the clinic prior to taking home the device. All subjects that succeeded in the clinical setting also succeeded in the simulated home environment. Using the mean of three measurements was advised in earlier guidelines [16], but this was recently changed to two measurements [13]. We found essentially the same agreement between the two devices when comparing the mean of three valid measurements in the NIOX and the first valid measurement in the NIOX MINO. We thus suggest that one measurement is adequate when using the NIOX MINO, which would save valuable time in the clinic. The time for NO analysis in the NIOX MINO is 100 s, but since one measurement seems to be adequate in most instances, the total measurement time will still be acceptable.

## Conclusion

The results show that there is clinically acceptable agreement between the stationary NIOX and the new hand-held NIOX MINO, when similar conditions were considered and examinations were made as consistently as possible. The repeatability of measurements done using the hand-held device was similar to the stationary device, and adults and most children were able to successfully use both instruments. In addition, subjects displayed ability to operate the new hand-held device in a simulated home-use environment. The new hand-held instrument will enable the introduction of exhaled NO measurements in the primary health care.

## Competing interests

KA is a co-founder and a minority shareholder of Aerocrine AB. LN is a minority shareholder of Aerocrine AB. CJ reports no conflict of interest.

## Authors' contributions

KA participated in the analysis of data and drafted the manuscript. CJ and LN participated in the design and coordination of the study. All authors read and approved the final manuscript.
